# High prevalence of Group B *Streptococcus* colonization among pregnant women in Amman, Jordan

**DOI:** 10.1186/s12884-019-2317-4

**Published:** 2019-05-20

**Authors:** Kate Clouse, Asem Shehabi, Abel Mani Suleimat, Samir Faouri, Najwa Khuri-Bulos, Abeer Al Jammal, James Chappell, Kimberly B. Fortner, Anna B. Chamby, Tara M. Randis, Adam J. Ratner, David M. Aronoff, Natasha Halasa

**Affiliations:** 1 Department of Medicine, Division of Infectious Diseases, Vanderbilt University Medical Center, Nashville, TN USA; 2 Vanderbilt Institute for Global Health, Nashville, TN USA; 3 University of Jordan, Amman, Jordan; 4 Al-Bashir Hospital, Amman, Jordan; 5 Department of Pediatrics, Vanderbilt University Medical Center, Nashville, TN USA; 6 Department of Obstetrics and Gynecology, Vanderbilt University Medical Center, Nashville, TN USA; 7 Departments of Pediatrics and Microbiology, New York University School of Medicine, New York, NY USA; 8 Department of Pathology, Microbiology, and Immunology, Vanderbilt University Medical Center, Nashville, TN USA

**Keywords:** Group B *Streptococcus*, GBS, Pregnancy, Jordan, Middle East

## Abstract

**Background:**

Little is known of the burden of Group B *Streptococcus* (GBS) colonization among pregnant women in Jordan. We conducted a pilot study to determine the prevalence of GBS among pregnant women in Amman, Jordan, where GBS testing is not routine. We also explored GBS serotypes and the performance of a rapid GBS antigen diagnostic test.

**Methods:**

We collected vaginal-rectal swabs from women who presented for labor and delivery at Al-Bashir Hospital. Three methods were used to identify GBS: Strep B Rapid Test (Creative Diagnostics), blood agar media (Remel) with confirmed with BBL Streptocard acid latex test (Becton Dickinson), and CHROMagar StrepB (Remel). Results were read by a senior microbiologist. We defined our gold standard for GBS-positive as a positive blood agar culture confirmed by latex agglutination and positive CHROMagar. PCR testing determined serotype information. Demographic and clinical data were also collected.

**Results:**

In April and May 2015, 200 women were enrolled with a median age of 27 years (IQR: 23–32); 89.0% were Jordanian nationals and 71.9% completed secondary school. Median gestational age was 38 weeks (IQR: 37–40); most women reported prenatal care (median 9 visits; IQR: 8–12). Median parity was 2 births (IQR: 1–3). Pre-pregnancy median BMI was 24.1 (IQR: 21.5–28.0) and 14.5% reported an underlying medical condition. Obstetric complications included gestational hypertension (9.5%), gestational diabetes (6.0%), and UTI (53.5%), of which 84.5% reported treatment. Overall, 39 (19.5%) of women were GBS-positive on blood agar media and CHROMagar, while 67 (33.5%) were positive by rapid test (36% sensitivity, 67% specificity). Serotype information was available for 25 (64%) isolates: III (48%), Ia (24%), II (20%), and V (8%). No demographic or clinical differences were noted between GBS+ and GBS-negative women.

**Conclusions:**

Nearly one in five women presenting for labor in Jordan was colonized with GBS, with serotype group III as the most common. The rapid GBS antigen diagnostic had low sensitivity and specificity. These results support expanded research in the region, including defining GBS resistance patterns, serotyping information, and risk factors. It also emphasizes the need for routine GBS testing and improved rapid GBS diagnostics for developing world settings.

## Background

Group B *Streptococcus* (*S. agalactiae; “*GBS”) is a gram-positive bacterium with a special capacity to cause perinatal infections of the mother, fetus, and/or newborn. This pathogen causes chorioamnionitis [1], preterm birth [2], stillbirth [3], meningitis [4] and is a leading cause of both early-onset (< 7 days of life) and late-onset (7–89 days of life) neonatal sepsis [5]. Globally, the burden of GBS disease is estimated to be 0.49–0.53 per 1000 livebirths, with a case fatality rate of 8.4–9.6% [6, 7]. The incidence of early-onset GBS disease is estimated to be 0.43 per 1000 livebirths, with a case fatality rate of 12.1%, twice that of late-onset disease [6].

In the United States, recommendations for routine screening for GBS in women between 35 and 37 weeks pregnant, followed by antibiotic prophylaxis 4h prior to delivery for colonized patients, led to a dramatic decrease in the incidence of early-onset GBS disease in neonates [8], with no change in the incidence of late-onset GBS. This screen-and-treat paradigm, however, has not been widely adopted outside of the United States, and policy decisions have been challenged by the absence of solid estimates of the number of at-risk mothers and babies in many parts of the world [9]. One barrier to obtaining reliable epidemiological data from low- and middle-income countries has been the absence of accurate point-of-care tests to determine GBS colonization without the need for time-consuming and expensive, laboratory-based cultivation practices [10].

A 2016 meta-analysis including data for over 70,000 women in 37 countries estimated global prevalence of maternal GBS colonization at 17.9% (95%CI: 16.2–19.7%) [11]. However, the authors noted substantial heterogeneity across and within regions, and reported few studies from Middle East and North African (MENA) countries, highlighting a paucity of maternal GBS prevalence data from the region. From our own review, we found many reports from the MENA region estimating the frequency of GBS in pregnant mothers, with estimates ranging from 1.6 to 32% (Table 1), however nearly half of these studies were conducted in only two countries – Iran and Israel – and over one-third of the studies were published more than 10 years ago. In Jordan, universal screening is not routine. Only one manuscript, published in 1991, estimated the GBS prevalence among 500 pregnant women in Jordan, finding nearly one-third (30.4%) of women were colonized by vaginal, rectal, and/or urine specimens [35]. Prior to launching future studies to assess novel diagnostics and vaccines, it is essential to establish the current burden of GBS colonization in the region. Given the scarcity of information about GBS burden of colonization in Jordan, we conducted a pilot study to determine the prevalence of GBS recto-vaginal colonization among pregnant women presenting for labor at one high-volume government hospital in Amman, Jordan, where GBS testing is not routine. Secondary objectives were to assess capsular serotypes of GBS specimens and to compare the performance of a GBS rapid diagnostic to culture.

**Table 1:** Estimates from Middle East and North Africa countries of Group B *Streptococcus* prevalence among pregnant women

Country	Year published	N	GBS proportion	First author
Egypt [12]	2017	80	11.3%	Wassef
Egypt [13]	2014	364	27.4%	Shabayek
Egypt [14]	2009	95	17.9%	Elbaradie
Egypt [15]	2009	150	25.3%	Shabayek
Iran [16]	2017	186	11.8%	Darabi
Iran [17]	2016	203	24.1%	Mousavi
Iran [18]	2016	237	12.6%	Sadeh
Iran [19]	2016	137	30.7%	Bidgani
Iran [20]	2015	100	17.0%	Goudarzi
Iran [21]	2015	210	3.3%	Hadavand
Iran [22]	2014	980	4.9%	Shirazi
Iran [23]	2013	1028	22.8%	Javanmanesh
Iran [24]	2013	285	9.5%	Tajbakhsh
Iran [25]	2012	200	6.0%	Hamedi
Iran [26]	2011	310	13.8%	Hassanzadeh
Iran [27]	2008	1197	9.1%	Namavar Jahromi
Israel [28]	2018	188	31.0%	Hakim
Israel [29]	2016	935	31.5%	Sefty
Israel [30]	2015	542	24.9%	Kabiri
Israel [31]	2015	116	18.1%	Ganor-Paz
Israel [32]	2006	629	13.7%	Eisenberg
Israel [33]	2003	681	12.3%	Marchaim
Israel [34]	1990	257, 189, 116	5.4, 1.6, 3.5%	Eidelman
Jordan [35]	1991	500	30.4%	Sunna
Kuwait [36]	2014	1391	20.7%	Ghaddar
Kuwait [37]	2005	847	14.6%	Al-Sweih
Lebanon [36]	2014	168	18.4%	Ghaddar
Lebanon [38]	2010	775	17.7%	Seoud
Morocco [39]	2018	350	24.0%	Moraleda
Morocco [40]	2016	275	20.2%	Bassir
Saudi Arabia [41]	2015	1328	13.4%	Khan
Saudi Arabia [42]	2011	326	31.6%	Zamzami
Saudi Arabia [43]	2002	217	27.6%	El-Kersh
Tunisia [44]	2007	294	12.9%	Jerbi
Tunisia [45]	2006	300	13.0%	Ferjani
Turkey [46]	2016	215	9.8%	Alp
Turkey [47]	2005	500	9.2%	Eren
Turkey [48]	2005	150	32.0%	Kadanali
Turkey [49]	2005	300	8.0%	Barbaros
UAE [50]	2002	563	10.1%	Amin
UAE [51]	2002	891	21.5%	Sidky

## Methods

### Study design and population

This was a 2-month cross-sectional study to determine the prevalence of GBS colonization among women admitted for labor and delivery at Al-Bashir Hospital in Amman, Jordan. Al-Bashir Hospital is one of three major government-run referral medical centers in Amman, which is Jordan’s largest city and capital. There are an estimated 1300 deliveries per month. Intrapartum antibiotic prophylaxis (IAP) for suspected GBS colonization was provided per clinician discretion based on maternal risk factors and other indications for suspected infection. Other intrapartum antibiotic provision was at clinician discretion.

This study was approved by the institutional review boards of Vanderbilt University Medical Center, University of Jordan, and the Jordan Ministry of Health. All participants provided written informed consent prior to enrollment. To maintain participant confidentiality, unique study identification numbers were used in lieu of personal identifiers.

### Data and specimen collection

After consent, trained local research staff interviewed the pregnant women using a standardized questionnaire to record maternal and paternal demographic characteristics, history of antenatal care, and medical and obstetric history. Subjects were queried in Arabic, and bilingual research staff transcribed the information onto an English-language case report form at the time of the interview. After subjects were discharged, charts were abstracted for maternal outcomes, antibiotic use, and length of stay.

GBS specimen collection and processing were conducted according to the 2010 US Centers for Disease Control and Prevention (CDC) recommendations [52]. A single combined vaginal-rectal swab was collected from each participant prior to delivery, placed into LIMBroth tubes, transported to a laboratory at the University of Jordan, placed in an incubator at 35–37 degrees Celsius, and then further sub-cultured after a minimum of 18h. Three methods were used to identify GBS. Point-of-care GBS testing was performed with Strep B Rapid Test (Creative Diagnostics), which was selected based on availability, cost, and ease of use. Two laboratory methods to identify GBS included blood agar media (Becton Dickinson) confirmed with BBL Streptocard acid latex test (Remel) and CHROMagar StrepB (Remel). Results were read by a senior microbiologist at the University of Jordan. We defined our gold standard for GBS-positive as a positive blood agar culture confirmed by both latex agglutination and positive CHROMagar.

Samples determined as GBS-positive were shipped from Jordan to the United States for additional confirmatory test on chromogenic agar and PCR of the conserved *sip* gene, as described [53]. About one-third (14/39, 35.9%) of the samples did not survive transport. For the 25 viable strains, GBS serotypes were determined using a nested PCR-based strategy [54] and confirmed with latex agglutination (IMMULEX™ STREP-B kit, Statens Serum Institut Diagnostica, Hillerød, Denmark).

Study data were collected and managed using REDCap electronic data capture tools hosted at Vanderbilt University [55], and analyzed using SAS, version 9.4 (SAS Institute, Inc., Cary, NC).

### Analysis

We present counts and proportions for categorical variables and medians and interquartile ranges (IQR) for continuous variables. Patient characteristics were compared by GBS outcome using Wilcoxon rank sums and Chi-square statistics to assess differences in medians and distributions, respectively. In the event of small cell sizes (*n* < 5), Fisher’s exact test was substituted for Chi-square.

## Results

### Patient characteristics

Overall, 226 women who presented in labor during April and May 2015 at Al-Bashir Hospital were approached; 26 women refused enrollment, for total cohort of 200 women. Demographic and clinical characteristics of the 200 participants are displayed in Table 2. The median participant age was 27 years (IQR: 23–32). Most participants were Jordanian nationals (89.0%), had completed secondary school (71.9%), and had not been employed in the past year (86.2%). Median gestational age at delivery was 38 weeks (IQR: 37–40). All but one woman (99.5%) reported attending antenatal care, and most made frequent visits (median 9 visits; IQR: 8–12). Median maternal pre-pregnancy BMI was 24.1 (IQR: 21.5–28.0); median gravidity was 3 pregnancies (IQR: 2–5), and parity was 2 births (IQR: 1–3). Cesarean-sections were common: overall, 96/200 (48.0%) deliveries were by cesarean; of these, 79/98 (80.6%) were repeat C-sections. Few women reported smoking cigarettes (8.5%) and narghile (tobacco hookah, 4.5%) during this pregnancy. However, most women reported living in a household with at least one cigarette smoker (63.0%) and some reported household narghile smoking (16.5%). No demographic or clinical differences were noted between GBS-positive and GBS-negative participants (Table 2).

**Table 2:** Characteristics of 200 participants tested for Group B Streptococcus in Amman, Jordan overall, and by confirmed GBS results

Participant characteristic	Overall (*n*=200)	Confirmed GBS (*n*=39)	No confirmed GBS (*n*=161)	*p*-value
Age, *median (IQR)*	27 (23-32)	28 (23-33)	27 (23-32)	0.83
Age, n (%)				
16-23 years	55 (27.5)	11 (28.2)	44 (27.3)	0.94
24-32 years	97 (48.5)	18 (46.2)	79 (49.1)	
33 years and older	48 (24.0)	10 (25.6)	38 (23.6)	
Education, *n (%)*				
Primary school or less	56 (28.1)	10 (25.6)	46 (28.8)	0.20
Secondary school	115 (57.8)	20 (51.3)	95 (59.4)	
Post-secondary school	28 (14.1)	9 (23.1)	19 (11.9)	
Nationality, *n (%)*				
Jordanian	177 (88.9)	35 (89.7)	142 (88.8)	0.86
Other	22 (11.1)	4 (10.3)	18 (11.3)	
Gestational age at delivery (weeks), *median (IQR)*	38 (37-40)	38 (37-40)	38 (37-40)	0.56
Antenatal visits, median	9 (8-12)	9 (9-12)	9 (8-12)	0.47
Pre-pregnancy BMI, *median (IQR*)	24.1 (21.5-28.0)	24.7 (21.5-29.4)	23.8 (21.4-27.9)	0.41
Gravidity, *median (IQR)*	3 (2-5)	3 (2-4)	3 (2-5)	0.52
Parity, *median (IQR)*	2 (1-3)	2 (1-3)	2 (1-3)	0.62

*p*-value testing the difference between participants with confirmed GBS (*n*=39) and without confirmed GBS (*n*=161)

Excludes missing values: education (*n*=1); nationality (*n*=1); BMI (*n*=1); gestational age (*n*=2); antenatal visits (*n*=1); BMI (*n*=1); parity (*n*=38)

Few underlying medical conditions were reported, most frequently high blood pressure (7.0%) and diabetes (3.0%). Obstetric complications during the current pregnancy reported included gestational hypertension (9.5%) and gestational diabetes (6.0%). Over half the women (53.5%) reported a urinary tract infection during the current pregnancy, of which 84.5% reported treatment (prior to the intrapartum period). Penicillin allergy was reported in 5.0% of all participants; no other drug allergies were reported.

### GBS colonization

In our cohort, 39/200 (19.5%) women were positive for GBS on both blood agar media with positive latex test and CHROMagar, our gold standard. The two tests used in our gold standard were 100% concordant. On the rapid antigen test, 67/200 (33.5%) were positive, of which, only 14/67 (21%) were confirmed GBS-positive by culture. There were 25 false-negative results and 53 false-positive results using the rapid test; sensitivity and specificity for the rapid test were 36 and 67%, respectively, compared to the gold standard of blood agar media with positive latex test and CHROMagar. The positive predictive value of the rapid test was 20.9%, the negative predictive value was 81.2%, the positive likelihood ratio was 1.09, and the negative likelihood ratio was 0.96.

### Antibiotic use

Data for intrapartum antibiotic prophylaxis (IAP) and antibiotic use were known for 190 subjects, and 43 (22.6%) of these subjects received an intrapartum antibiotic. Of the 39 women who were GBS-positive by culture, only nine (23.1%) received IAP; 5/9 (55.6%) of whom also had a positive rapid result. One-quarter of women who received an intrapartum antibiotic were GBS-positive by rapid test but not culture (11/43, 25.6%). Reasons for intrapartum antibiotic use are shown in Table 3.

**Table 3:** Intrapartum antibiotic prophylaxis (IAP) and antibiotic use, class of antibiotic, and GBS test results by confirmed GBS

	GBS-negative subjects (*n*=157)	GBS-positive subjects (*n*=39)	*p-value*
Intrapartum antibiotics use, *n (%)*^a^	34 (23.1)	9 (23.1)	*0.94*
GBS prophylaxis	6 (4.0)	4 (10.3)	0.12
C-section prophylaxis	16 (10.6)	4 (10.3)	1.00
UTI	3 (2.0)	2 (5.1)	0.27
Prolong rupture of membranes	3 (2.0)	0 (0.0)	1.00
Suspected amnionitis/chorioamnionitis	2 (1.3)	0 (0.0)	1.00
Rapid GBS-positive known	11 (7.2)	5 (12.8)	0.33
Rapid-positive for GBS, *n (%)*	53 (32.9)	14 (35.9)	0.72

^a^Missing antibiotic data for 10 GBS-negative participants; total n for this sections = 190

Multiple responses were possible for reason for antibiotic use; thus rows sum to greater than total

### Serotyping analysis

**Fig. 1:**
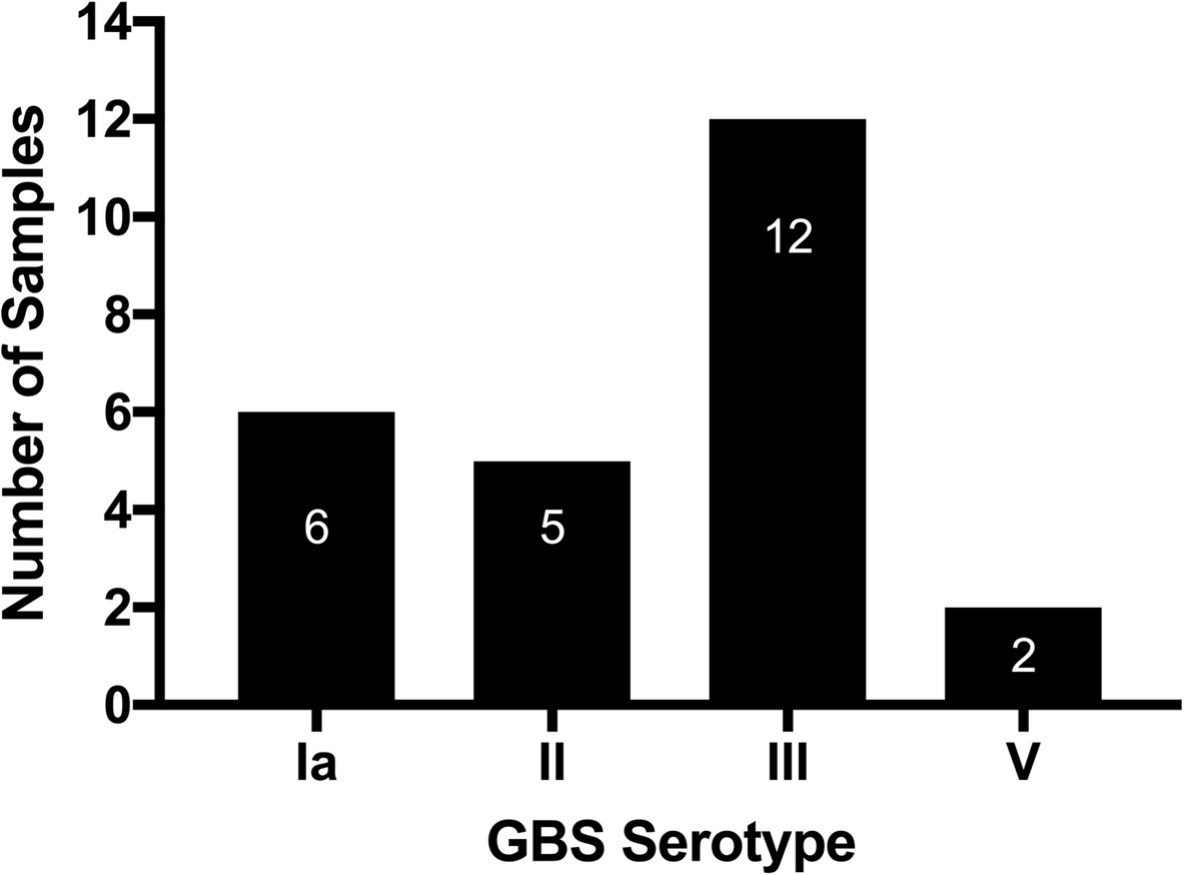
Group B *Streptococcus* serotypes (*n* = 25) identified among pregnant women in Amman, Jordan

Serotyping data are displayed in Fig. 1. The most prevalent GBS serotype identified was Type III (12/25, 48%), followed by Type Ia (6/25, 24%) and Type II (5/25, 20%). Only 8% (2/25) of cases were Type V. There were no Type Ib or Type IV GBS isolates identified.

## Discussion

Our surveillance study revealed nearly one in five women presenting for labor at Al-Bashir Hospital in Jordan were colonized with GBS. This represents a substantial burden of colonization within a context of no routine screening for GBS among pregnant women. Our findings are consistent with a worldwide-adjusted estimate for maternal GBS colonization of 18%, but a higher prevalence compared to Southern Asia (10%) and Eastern Asia (9.1%) [9]. However, our prevalence estimate is lower than the only other local data from Jordan, which reported 30.4% GBS colonization [35]. That study enrolled 500 women and included a positive GBS urine culture, rectal swab, and/or vaginal swab as part of their GBS colonization definition, but these data are nearly 30 years old [35]. The frequency of GBS colonization we found is average when compared to other countries in the MENA region (Table 1), where the average was 16.9% among 18,805 total subjects, with minimum of 1.6% and peak of 32% of subjects with GBS colonization. Thus, our study highlights that GBS colonization is common and potentially an important cause of neonatal disease. While our study did not include neonatal data, a 2017 study exploring neonatal sepsis at a tertiary hospital in Jordan found that GBS was isolated from 10% of neonatal sepsis cases; however, the authors did not explore overall prevalence of GBS among pregnant women [56]. Therefore, our study emphasizes that routine testing for GBS in mothers would be valuable in Jordan so IAP could be introduced to prevent early-onset GBS neonatal disease.

Based on clinical trials and observational studies, early-onset GBS disease can be prevented by the administration of IAP during labor to GBS colonized women, with potential efficacy of 80% [57, 58]. Therefore, two screening methods have been implemented to identify GBS antenatally: risk-based or universal culture-based screening between 35 and 37 weeks. In 2002, CDC recommended switching to culture-based universal screening. Subsequently, an uptake of prenatal screening and IAP was rapid and widespread [8]. In addition, the incidence of invasive early-onset GBS disease decreased by more than 80% from 1.8 cases/1000 live births in the early 1990s to 0.26 cases/1000 live births in 2010, estimating that over 70,000 cases of early-onset GBS invasive disease were prevented in the United States [8]^.^ However, globally, the majority of countries that routinely screen for GBS are risk-based, and unfortunately very few of these countries are from the MENA area, including Jordan which does not routinely test for GBS colonization. In our study, only 9/39 (23.1%) women with confirmed GBS received an intrapartum antibiotic; five of these were also diagnosed by rapid test.

There are no established international standards for sampling GBS colonization; however, CDC recommends rectovaginal swabs at 35–37 weeks with selective enrichment broth culture, but this approach is not always feasible for low and middle-income settings [9]. Additional barriers to routine testing in these low resource settings may include the lack of microbiology capacity to perform routine testing for GBS, lack of timely knowledge of the results being reported back to the clinicians because of separate settings where prenatal care is performed and where lab testing would be performed, and lack of electronic medical records for the obstetricians to obtain antenatal results. One way to address these issues is the introduction of point-of-care testing when women present in labor. Unfortunately, the rapid diagnostic used in this pilot study was substantially less sensitive and specific than culture. Furthermore, over one-quarter of women who received an intrapartum antibiotic due to a positive rapid GBS antigen test (11/43, 25.6%) were found later to be GBS culture-negative, leading to unnecessary use of antibiotics. Our GBS antigen test results were poorer than other studies using other antigen-based tests that yielded 57.3% sensitivity and 99.5% specificity in Canada [59] and 100% sensitivity and 92.9% specificity in India [60]. Other point-of-care testing methods such as PCR testing have been reported to have high sensitivity (92.9–100%) and specificity (81.1–97.5%) [19, 61, 62] and may be a better solution. However, these higher-performing tests typically are cost-prohibitive for lower-resource settings [12, 63], and further development of affordable point-of-care GBS tests are urgently needed, particularly those that also simultaneously provide information about antibiotic sensitivities.

GBS has 10 known serotypes; however, five (Ia, Ib, II, III, and V) colonize the rectovaginal tracts of women in all regions, accounting for 98% of serotypes globally [9]. Moreover, these five serotypes represent 97% of invasive isolates in all regions with serotype data [7], with serotype III the most prevalent serotype across the United Nations sub-regions. Our study also identified serotype III as the most prevalent, followed by Ia and II serotypes. This is similar to other studies that report serotype III as the first- or second-most common isolate colonizing women [64]. Knowing which serotypes are most widespread will be important when GBS conjugate vaccines become available [65]. Therefore, further studies are needed to know which serotypes are responsible for both colonization and invasive neonatal disease in the MENA region, including Jordan.

Our results should be viewed in light of a number of limitations. This study was conducted at one facility – a government hospital – over 2 months and may not be generalizable to other settings in Jordan or the MENA region. However, the frequency of GBS colonization is similar to the average reported for all MENA regions. Furthermore, we are unable to report neonatal outcome information and our use of one senior microbiologist may have affected our results. In addition, we did not test for antibiotic susceptibility, which is important considering reports of increasing erythromycin and clindamycin resistance in GBS [66]. Still, given the dearth of information about GBS in Jordan and the wider MENA region, our results help to shed light on the high prevalence of GBS colonization among pregnant women.

## Conclusions

We found high prevalence of GBS and both under- and over-treatment of GBS among pregnant women in Jordan. These results highlight the unmet need for routine GBS testing during pregnancy and support expanded research in the region, including defining the GBS resistance patterns, serotyping information, risk factors, and neonatal outcomes. They also emphasize the need for improved rapid GBS diagnostics for developing world settings.
